# Supplementary material for: Geographic Distribution Pattern Determines Soil Microbial Community Assembly Process in Acanthopanax senticosus Rhizosphere Soil
Source: Microorganisms. 2024 Dec 4;12(12):2506. doi: 10.3390/microorganisms12122506 (PMC11728389; doi:10.3390/microorganisms12122506)
Supplement: Supplementary file 1 [file microorganisms-12-02506-s001.zip › microorganisms-3314614-supplementary.pdf]

Table S1. Biomass of plant (*Acanthopanax senticosus*) at different sites.

Site	Root length (cm)	Stem length (cm)	Height (cm)	Annual branch thickness (cm)	Stem thickness (cm)	Chlorophyll content	Leaves number	Leaf length (cm)	Leaf width (cm)	Leaf area (cm²)	Branch number	Annual branch length (cm)
AC	12.6±1.386b	6.05±0.5542c	7.31±1.2684b	0.637±0.0846ab	1.598±0.2201b	26.26±2.1250bc	18.90±2.9608bc	5.47±0.9405b	2.49±0.4840c	11.55±1.0367c	6.8±1.3984a	6.92±1.3464b
FZ	13.03±2.018b	7.61±0.5342b	5.76±0.8996d	0.576±0.0931b	1.425±0.3007b	25.71±2.0420c	25.20±2.0439a	5.54±0.4903b	3.21±0.3928ab	12.63±1.2747bc	6.6±1.0749a	5.97±0.7498b
MS	8.18±0.621c	3.47±0.6832d	6.41±1.6285bc	0.642±0.1791ab	1.505±0.1735b	25.94±0.8382bc	19.10±3.7252b	8.08±1.1717a	3.67±0.6992a	18.25±5.3021a	6.6±0.8432a	6.39±1.1751b
MIS	8.85±0.7382c	5.18±0.5788c	7.36±1.2491b	0.626±0.0705ab	1.552±0.2226b	26.44±1.9631bc	15.6±3.6878c	5.67±1.1528b	3.21±0.8184ab	15.34±3.1473ab	6.7±1.2516a	7.06±0.9651b
QA	8.37±1.041c	5.77±0.5945c	5.72±0.8495d	0.683±0.1298ab	1.622±0.1127b	28.07±2.4931ab	19.90±2.6436b	5.36±0.8435b	2.61±0.5216bc	10.88±2.0283c	6.3±1.0593b	6.20±1.0519b
YC	18.18±1.813a	9.96±0.4708a	10.01±1.4502a	0.738±0.0820a	2.650±0.3373a	30.09±2.4973a	20.30±2.8693b	6.33±0.8705b	2.96±0.5420bc	10.68±2.5517c	6.7±1.0593a	8.56±1.6880a

Table S2. Plant root secondary metabolites at different sites.

Site	Chlorogenic acid (mg g^{-1})	Hyperoside (mg g^{-1})	Quercetin (mg g^{-1})	Syringin (mg g^{-1})
AC	0.1736±0.00554f	0.0065±0.00108c	0.0130±0.00149cd	0.0191±0.00281b
FZ	0.3818±0.08400a	0.0106±0.00201b	0.0112±0.00199de	0.0220±0.00287ab
MS	0.2133±0.00811e	0.0076±0.00165c	0.0134±0.00143c	0.0212±0.00204ab
MIS	0.2720±0.00631c	0.0107±0.00193b	0.0160±0.00163a	0.0207±0.00309ab
QA	0.2333±0.01379d	0.0169±0.00166a	0.0095±0.00184e	0.0224±0.00250a
YC	0.3445±0.00938b	0.0110±0.00183b	0.0146±0.00222ab	0.0155±0.00276c

Table S3. PERMANOVA analysis of soil bacterial communities, soil fungal communities, and functional predictions of soil bacteria and fungi.

OTU								Prediction of key functional genes							
Soil bacterial community				Soil fungal community				Soil bacterial community				Soil fungal community			
pairs	R2	p.value	p.adjusted	pairs	R2	p.value	p.adjusted	pairs	R2	p.value	p.adjusted	pairs	R2	p.value	p.adjusted
Group	0.594057698	0.001	0.001	Group	0.564022060	0.001	0.001	Group	0.276309154	0.001	0.005	Group	0.612016673	0.001	0.005
MIS vs FZ	0.575023772	0.001	0.001	MIS vs FZ	0.29259988	0.001	0.001	MIS vs FZ	0.413019817	0.001	0.001	MIS vs FZ	0.319191338	0.001	0.001
MIS vs QA	0.781055547	0.001	0.001	MIS vs QA	0.630827227	0.001	0.001	MIS vs QA	0.776152558	0.001	0.001	MIS vs QA	0.76892098	0.001	0.001
MIS vs AC	0.552931272	0.001	0.001	MIS vs AC	0.374440129	0.001	0.001	MIS vs AC	0.322449206	0.001	0.001	MIS vs AC	0.533889511	0.001	0.001
MIS vs MS	0.665435074	0.001	0.001	MIS vs MS	0.36194495	0.001	0.001	MIS vs MS	0.557527666	0.051	0.001	MIS vs MS	0.229327698	0.002	0.002
MIS vs YC	0.503511524	0.001	0.001	MIS vs YC	0.465926332	0.001	0.001	MIS vs YC	0.154156294	0.001	0.001	MIS vs YC	0.488280554	0.001	0.001
FZ vs QA	0.401203076	0.001	0.001	FZ vs QA	0.53975602	0.001	0.001	FZ vs QA	0.344063605	0.001	0.001	FZ vs QA	0.65282272	0.001	0.001

					3								6		
FZ vs AC	0.324152967	0.001	0.001	FZ vs AC	0.275684222	0.001	0.001	FZ vs AC	0.258096598	0.002	0.002	FZ vs AC	0.436289007	0.001	0.001
FZ vs MS	0.272173928	0.001	0.001	FZ vs MS	0.211190074	0.001	0.001	FZ vs MS	0.321948783	0.064	0.004	FZ vs MS	0.101846481	0.063	0.063
FZ vs YC	0.159218574	0.002	0.002	FZ vs YC	0.331240426	0.001	0.001	FZ vs YC	0.085702335	0.001	0.001	FZ vs YC	0.244699851	0.001	0.001
QA vs AC	0.682326598	0.001	0.001	QA vs AC	0.627448365	0.001	0.001	QA vs AC	0.698288482	0.001	0.001	QA vs AC	0.607470766	0.001	0.001
QA vs MS	0.598543106	0.001	0.001	QA vs MS	0.634284457	0.001	0.001	QA vs MS	0.317270374	0.001	0.001	QA vs MS	0.669046113	0.001	0.001
QA vs YC	0.309057497	0.001	0.001	QA vs YC	0.555852499	0.001	0.001	QA vs YC	0.1483952	0.001	0.001	QA vs YC	0.638706625	0.001	0.001
AC vs MS	0.394160376	0.001	0.001	AC vs MS	0.353177958	0.001	0.001	AC vs MS	0.305475396	0.002	0.002	AC vs MS	0.420426697	0.001	0.001
AC vs YC	0.355936344	0.001	0.001	AC vs YC	0.459599932	0.001	0.001	AC vs YC	0.113820564	0.001	0.001	AC vs YC	0.355124028	0.001	0.001
MS vs YC	0.30238	0.001	0.001	MS vs	0.422	0.001	0.001	MS vs	0.137320	0.001	0.001	MS vs	0.327	0.001	0.00

805	YC	52487<br>4	YC	73	YC	13389	1

Table S4. Topological parameters of soil bacterial and fungal networks in different sites.

Microbial community	Sample	Node number	Edge number	Average degree	Average weighting	Network diameter	Graph density	Modularity Index	Statistical inference
Bacteria	AC	34	21	1.235	2.4	3	0.037	0.902	148.467
	FZ	171	198	2.316	4.409	11	0.014	0.813	1212.424
	MS	189	178	1.884	3.584	15	0.01	0.893	1263.407
	MIS	177	200	2.26	4.306	11	0.013	0.861	1214.783
	QA	17	10	1.176	2.298	1	0.074	0.84	62.964
	YC	281	350	2.491	4.703	13	0.009	0.861	2191.138
Fungi	AC	21	11	1.048	2.072	2	0.052	0.893	79.943
	FZ	43	22	1.023	2.026	2	0.024	0.95	191.791
	MS	19	10	1.053	2.082	2	0.058	0.88	70.621
	MIS	16	8	1	1.975	1	0.067	0.875	56.424
	QA	10	5	1	1.975	1	0.111	0.8	30.899
	YC	6	3	1	1.986	1	0.2	0.667	15.579
Bacteria×Fungi	AC	179	2113	23.609	22.716	7	0.133	0.395	5909.158
	FZ	49	41	1.673	1.65	6	0.035	0.846	246.993
	MS	158	544	6.886	6.706	17	0.044	0.595	2037.474
	MIS	152	466	6.132	5.963	15	0.041	0.659	1813.496
	QA	56	48	1.714	1.633	5	0.031	0.842	292.456
	YC	69	72	2.087	1.976	5	0.031	0.85	382.466

A Node is the basic element in a network diagram, and in microbial network analysis, it represents a species or OUT. Node number indicates the number of nodes in the network. Edges are the connecting lines between nodes and represent the relationship or connection between nodes. Edge number indicates the number of edges in the entire network. The average degree is the sum of the degrees of all nodes in the network divided by the number of nodes is the average degree, which reflects the average interaction strength of the microorganisms in the network. Modularity index refers to the number and structure of modules made up of nodes in a network. Networks with a high modularity have a distinct substructure, whereas networks with a low modularity have

few substructures. Network Density is the ratio of the number of edges actually present in the network to the maximum hypothetical number of edges. The average weighting in a co-occurrence network is the average of the weights of all edges in the network, and the average weight is a metric describing the average strength of the association between nodes in the network.

Table S5. For Redundancy Analysis (RDA), the coefficient of determination (r^2) quantifies how well the explanatory variables explain the variability in the response variable, and the P-value evaluates the statistical significance of the model (or hypothesis). (The relationship between soil microbial communities and soil chemical properties)

	Soil bacterial community		Soil fungal community	
Soil chemical properties	r^2	Pr (>r)	r^2	Pr (>r)
pH	0.557541395	0.001	0.169042485	0.004
TN	0.211345215	0.002	0.12417261	0.013
TP	0.473087075	0.001	0.048833019	0.248
TK	0.057178829	0.202	0.104531913	0.047
EC	0.149401732	0.008	0.462176888	0.001
CEC	0.04327988	0.31	0.062521114	0.153
SOC	0.297627437	0.001	0.09867623	0.049

Table S6. For Redundancy Analysis (RDA), the coefficient of determination (r^2) quantifies how well the explanatory variables explain the variability in the response variable, and the P-value evaluates the statistical significance of the model (or hypothesis). (The interplay between soil microbial communities and plant biomass)

	Soil bacterial community		Soil fungal community	
Plant biomass	r^2	Pr(>r)	r^2	Pr(>r)
root length	0.145285693	0.009	0.459373531	0.001
stem length	0.168665068	0.019	0.200271657	0.007
height	0.129644846	0.022	0.276453003	0.002
annual branch thickness	0.028873465	0.418	0.01170719	0.74
stem thickness	0.181344284	0.003	0.161130594	0.017
chlorophyll content	0.169510083	0.004	0.047361219	0.232
number of leaves	0.118504376	0.026	0.026744374	0.46
leaf length	0.114414281	0.035	0.066343529	0.151
leaf width	0.006896044	0.793	0.101296295	0.055
leaf area	0.059589259	0.146	0.018516021	0.571
branch number	0.004667483	0.936	0.088252764	0.077
annual branch length	0.078856657	0.087	0.163779014	0.008

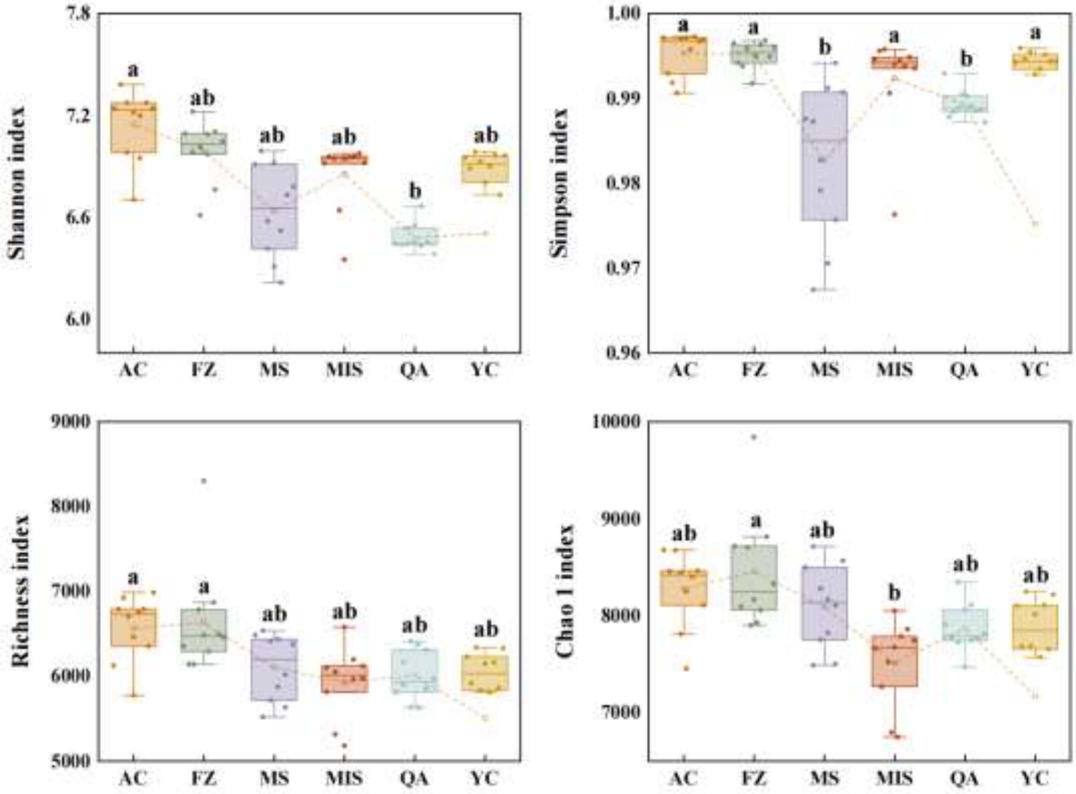

Figure S1. The diversity indices Shannon (a), Simpson (b), Richness (c) and Chao1 (d) of soil bacterial communities at the different sites. Different lowercase letters indicate significant differences in soil bacterial communities between the sites at the 5% level ($P < 0.05$) as analyzed by ANOVA and Tukey's post-hoc test.

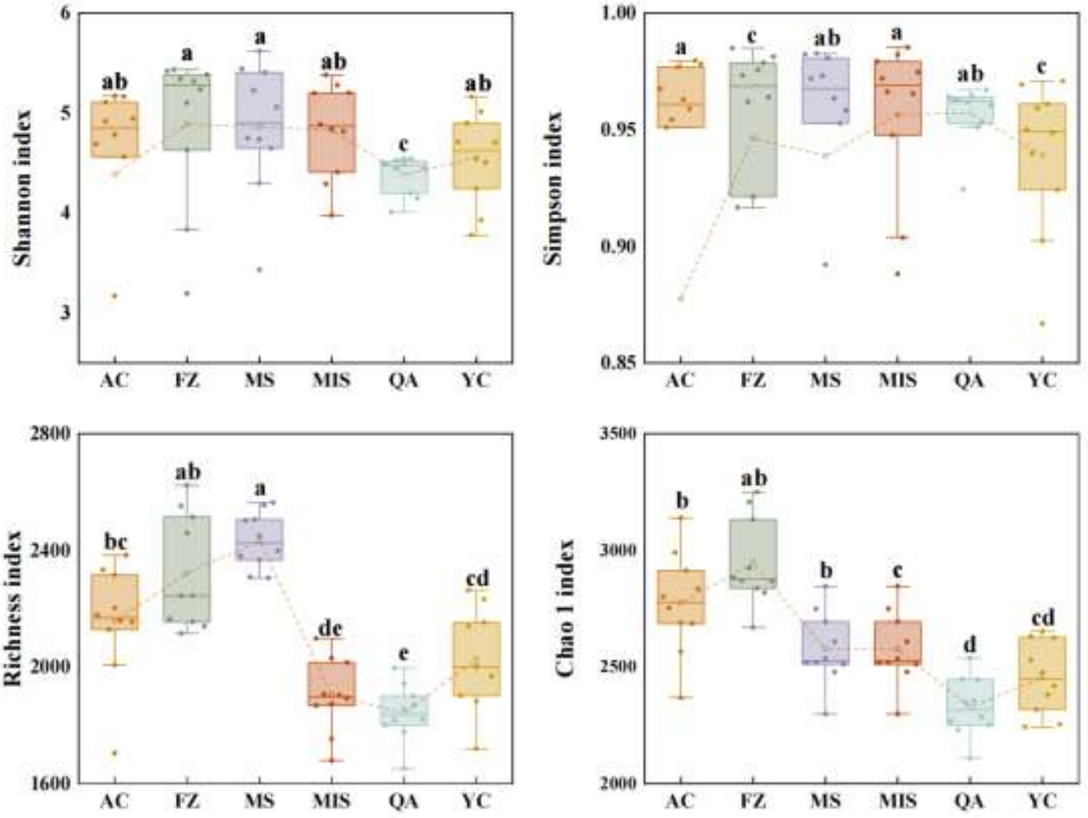

Figure S2. The diversity indices Shannon (a), Simpson (b), Richness (c) and Chao1 (d) of soil fungal communities at the different sites. Different lowercase letters indicate significant differences in soil bacterial communities between the sites at the 5% level (P < 0.05) as analyzed by ANOVA and Tukey's post-hoc test.

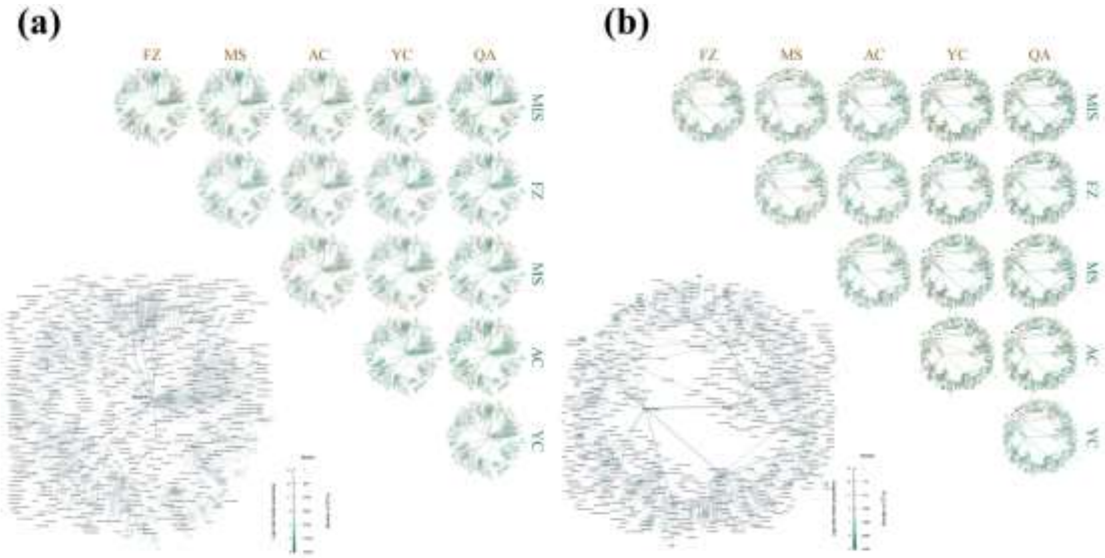

Figure S3. Heat tree showing average percentage of taxonomically evolved species of bacteria and fungi. The nodes on these trees denote each taxonomic level, progressing from kingdom (bacteria at the center) to species (located at the ends of each branch). Both the node and the edge (or branch) width reflect the average percentage of that taxon among samples within that specific group. The size of the nodes corresponds to the taxon count, while color intensity signifies their relative proportion in bacterial samples overall. Each taxon's color signifies the $\log^{-2}$ ratio of the median read proportions detected at various body sites. Only notable differences, determined through a Wilcox rank-sum test followed by a Benjamini-Hochberg (FDR) adjustment for multiple comparisons, are shaded. The gray tree in the bottom left corner serves as a reference for the smaller, unlabeled trees. Taxa shaded in green are prevalent in the soil microbial section indicated by the row, while those in brown are abundant in the soil microbial section denoted by the column. a: bacteria; b: fungi.

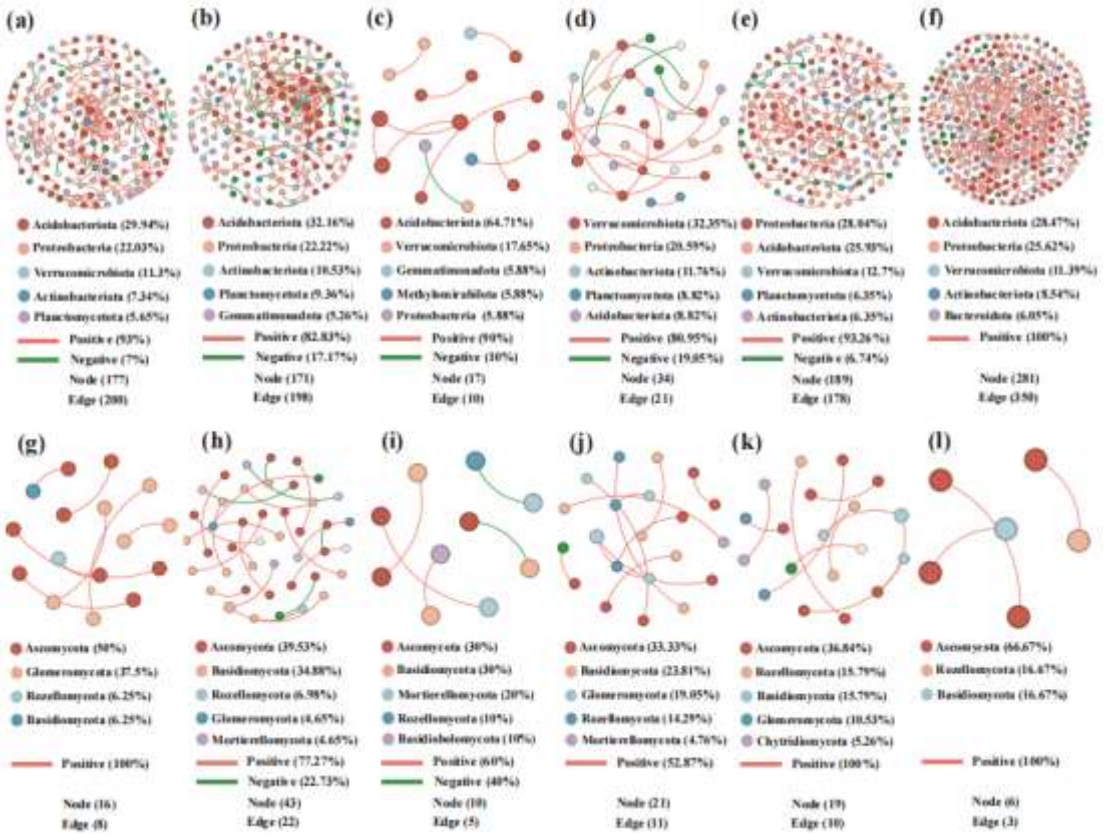

Figure S4. Co-occurrence network showing the network complexity of soil bacterial (a-f) and fungal (g-i) communities in the different sites. Dots of different colors represent different bacterial or fungal phyla. Lines represent correlations at a significance level of ($P < 0.05$), categorized as positive edge (Spearman's $\rho > 0.6$; red) or negative edge (Spearman's $\rho < -0.6$; green). a, g: AC; b, h: FZ; c, i: MS; d, j: MIS; e, k: QA; f, l: YC.

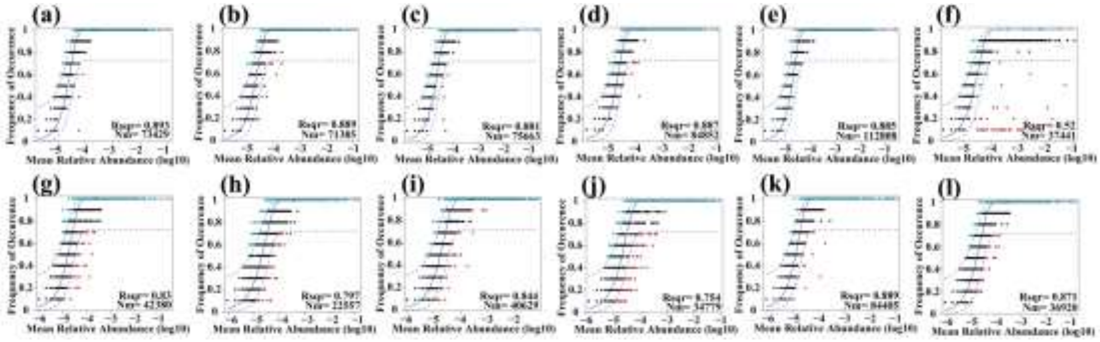

Figure S5. Neutral community models (NCM) fitting of soil bacterial and fungal communities in the soil samples. R^2(Rsqr) indicates the degree of fit of the model. Higher R^2 values indicate an assembly closer to a neutral model, where community construction is more influenced by stochastic processes and less by deterministic processes. Nm is the product of the metacommunity size N and mobility (m); Nm is used to quantify estimates of dispersal among communities and to determine the correlation between frequency of occurrence and relative abundance of the region. The solid blue line gives the best fit to the NCM, and the dashed blue lines indicate the 95% confidence interval around the NCM predictions, with OTUs (black dots) within the confidence interval considered to be neutrally distributed. a-f: bacteria; g-i: fungi. a, g: AC; b, h: FZ; c, i: MS; d, j: MIS; e, k: QA; f, l: YC.

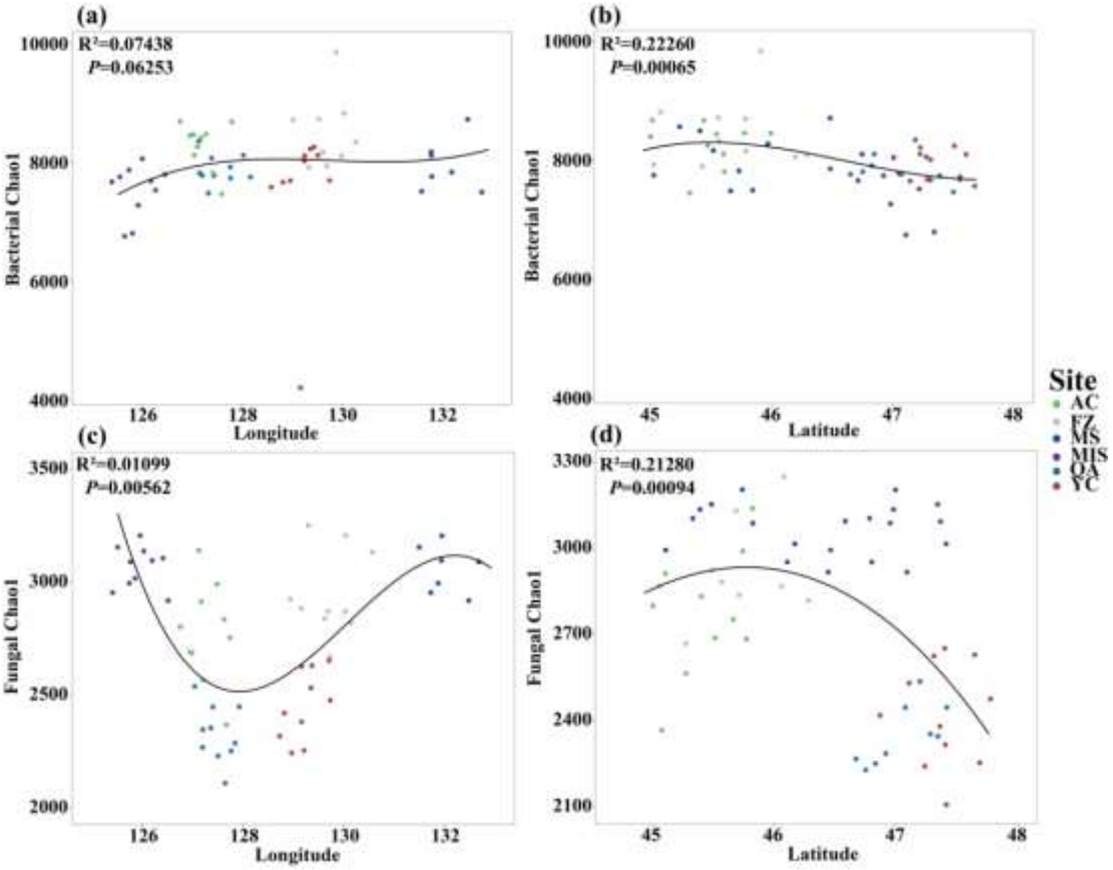

Figure S6. Nonlinear fit of geographic location of the sampled sites and the Chao1 index of soil bacterial (a, b) and fungal (c, d) communities, with longitude shown in a and c, and latitude in b and d.

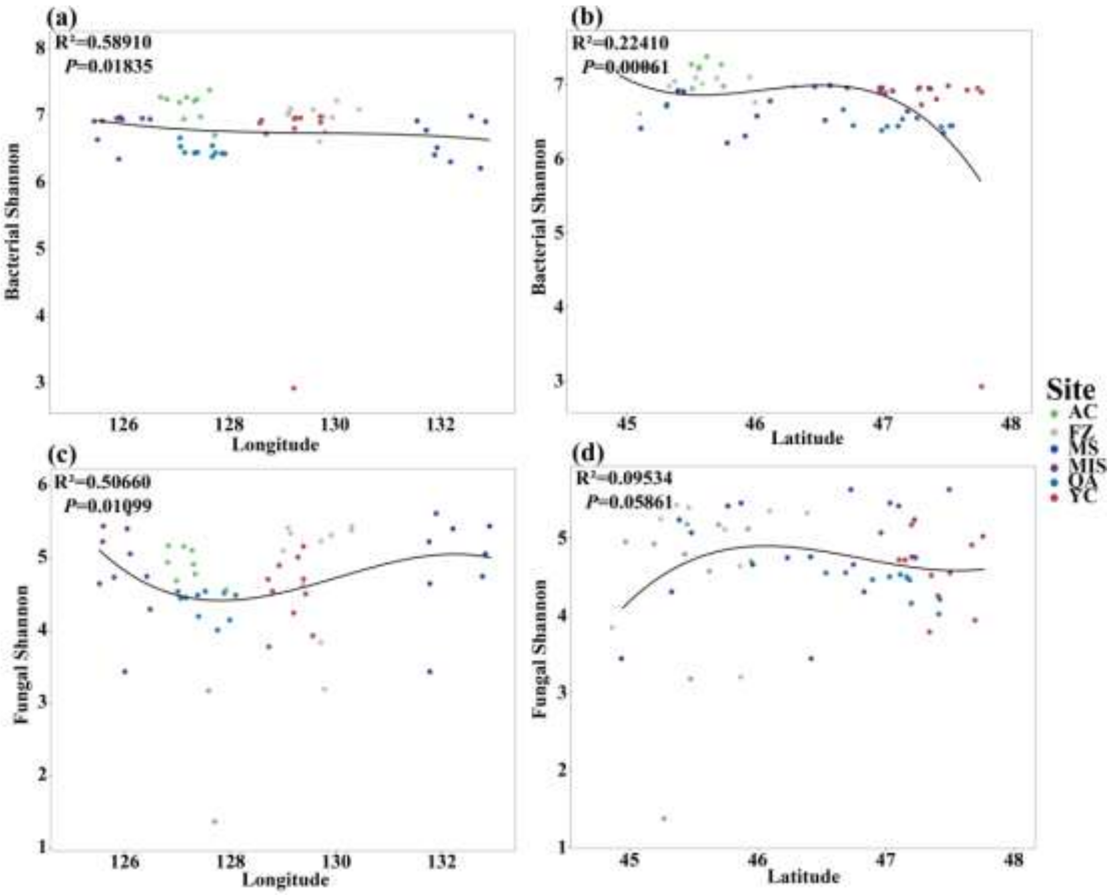

Figure S7. Nonlinear fit of geographic location of the sampled sites and the Shannon index of soil bacterial (a, b) and fungal (c, d) communities, with longitude shown in a and c, and latitude in b and d.

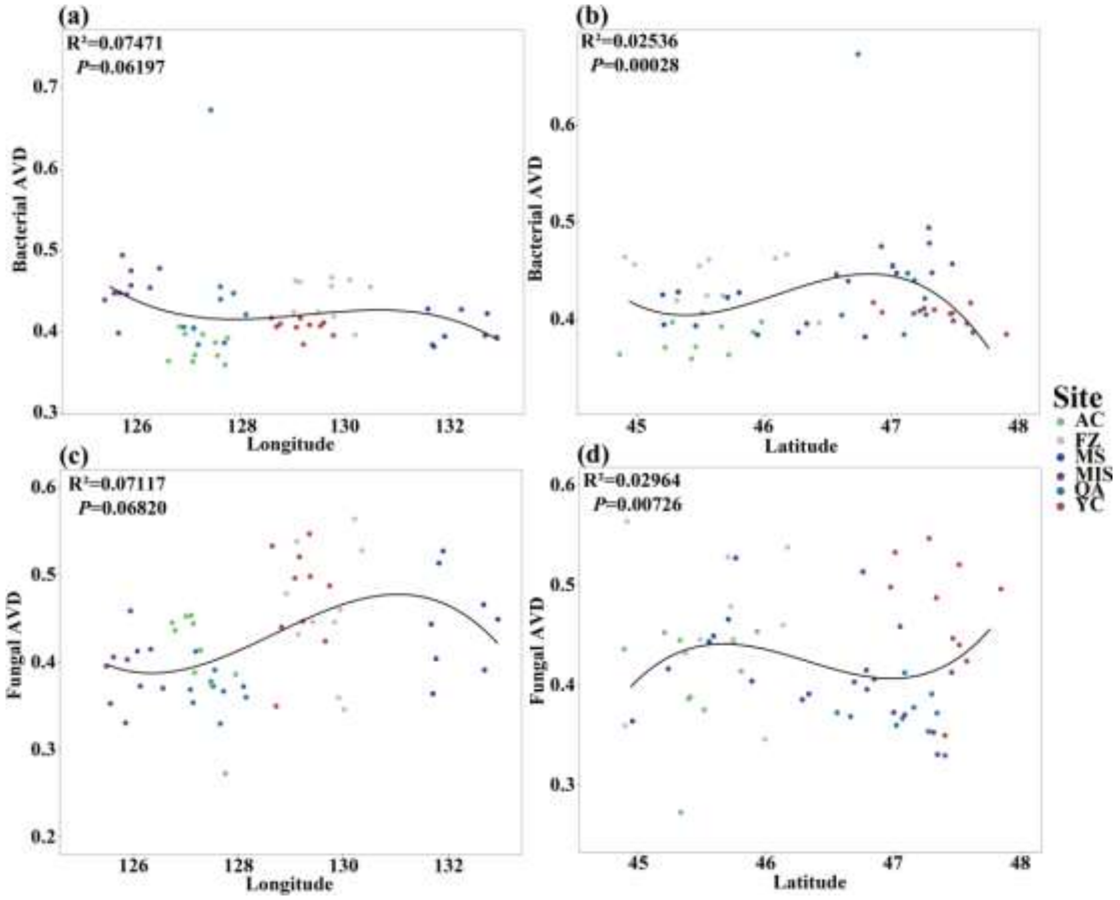

Figure S8. Nonlinear fit of geographic location of the sampled sites and the AVD index of soil bacterial (a, b) and fungal (c, d) communities, with longitude shown in a and c, and latitude in b and d.

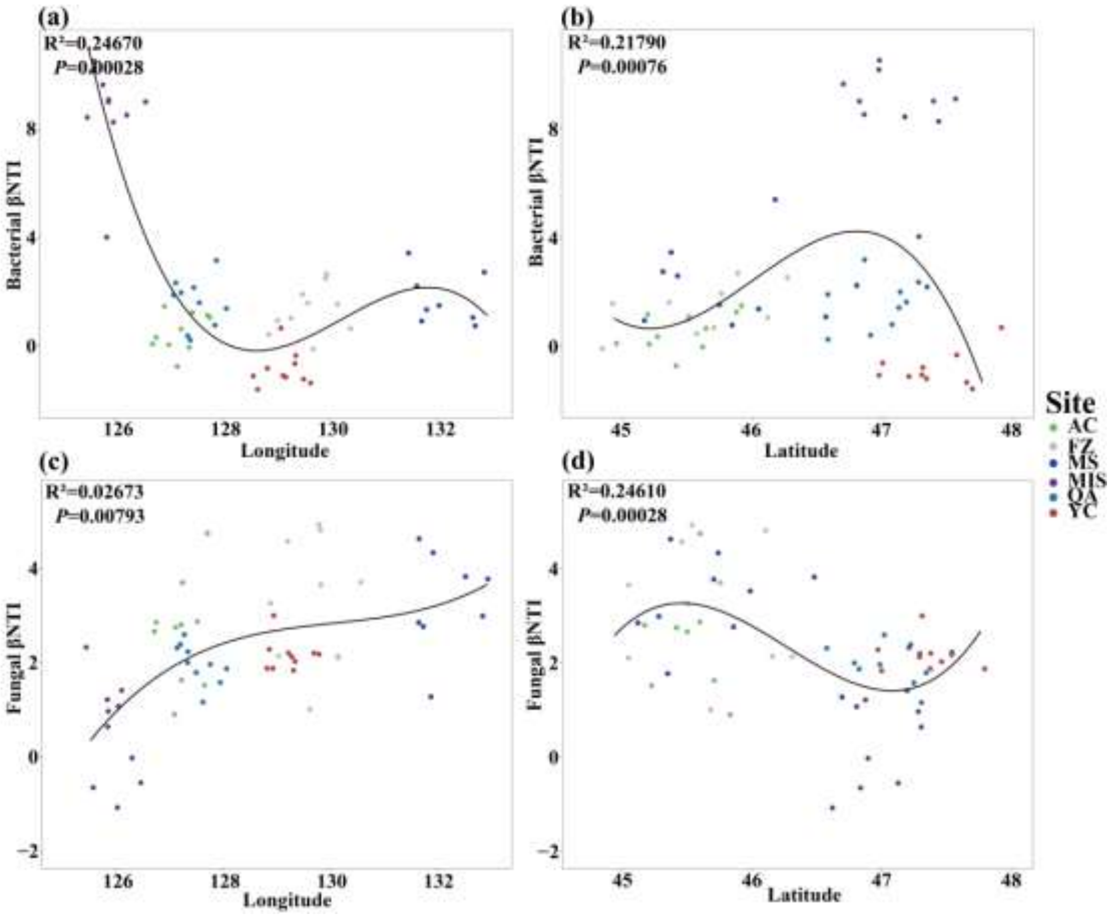

Figure S9. Nonlinear fit of geographic location of the sampled sites and the βNTI index of soil bacterial (a, b) and fungal (c, d) communities, with longitude shown in a and c, and latitude in b and d.

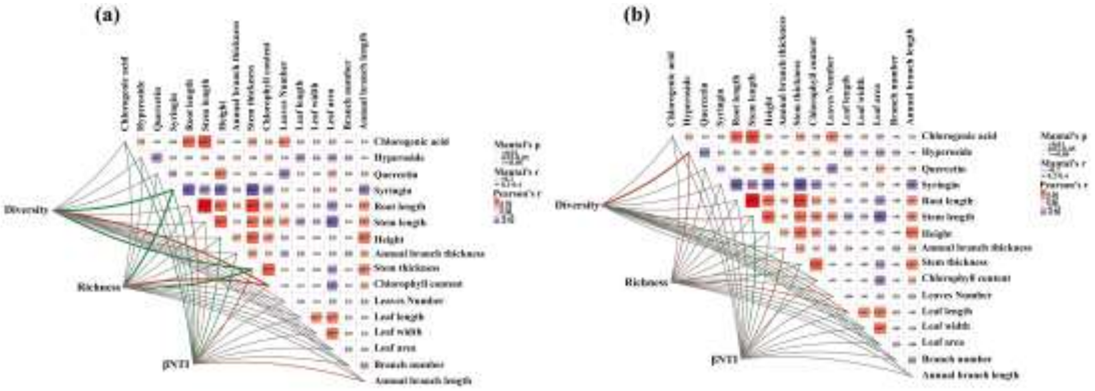

Figure S10. Mantel analysis demonstrating the relationship between soil bacterial (a) and fungal (b) communities Diversity, Richness and βNTI index with Plant root secondary metabolites and plant biomass. The red and green lines in figure c and d represent correlations, respectively, and a gray line represents no correlation. The thickness of the line represents the magnitude of the correlation (as per Spearman’s correlation coefficient).

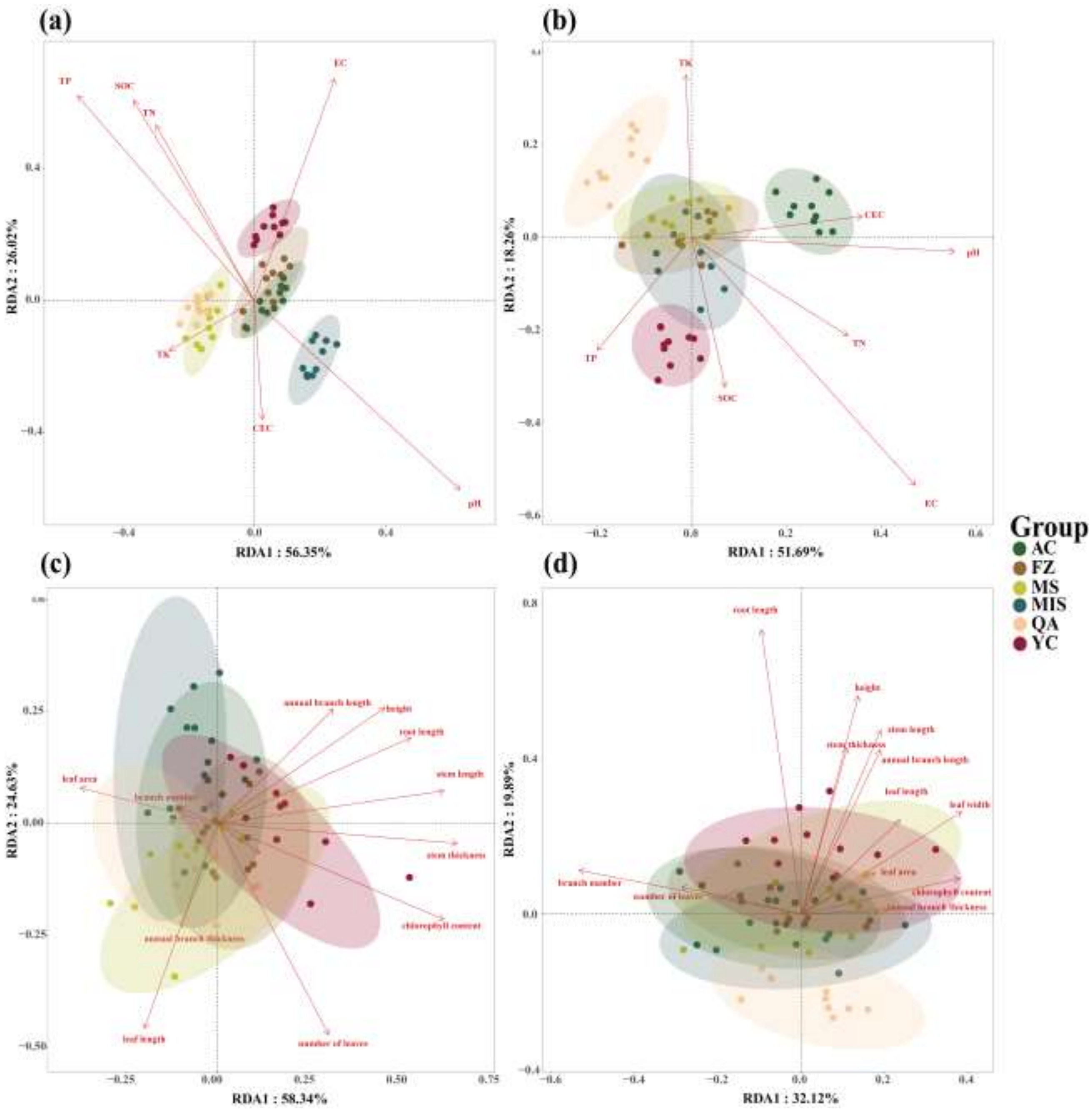

Figure S11. Redundancy analysis (RDA) of the relationships in soil bacterial (a) and fungal (b) communities composition and plant biomass under different sites. Redundancy analysis (RDA) of the relationships in soil bacterial (c) and fungal (d) communities and plant biomass under different sites. The closer the distance between two dots, the higher the functional similarity of the two samples. The longer the ray, the greater the influence of the factor on the structure and function of the colony; the angle between the arrow ray and the coordinate axis represents the size of the correlation between a certain environmental factor and the coordinate axis; the smaller the angle, the higher the correlation; the position of the sample projection point on the blue arrow: an approximate representation of the size of the value of the factor in the corresponding sample. Percentage next to the axes represents the proportion of the variance in the raw data that can be explained by the corresponding axes.
